# Influence of Lipstick Application on the Attractiveness of Smile in Women With and Without Malocclusions: An Eye‐Tracking Study

**DOI:** 10.1111/ocr.12947

**Published:** 2025-06-07

**Authors:** Gil Guilherme Gasparello, Mohamad Jamal Bark, Giovanna Purkote Yamaguchi, Rosilene Andrea Machado, Joel Suárez, Orlando Tanaka

**Affiliations:** ^1^ University of Oulu Oulu Finland; ^2^ School of Medicine and Life Sciences Pontifícia Universidade Católica Do Paraná Curitiba Brazil; ^3^ School of Life Sciences Pontifícia Universidade Católica Do Paraná Curitiba Brazil; ^4^ PUCRS Porto Alegre Brazil; ^5^ ABO‐PR Curitiba Brazil; ^6^ Universidade Técnica de Oruro Oruro Bolivia

**Keywords:** Eyetracking, malocclusion, orthodontics, perception

## Abstract

**Introduction:**

The use of cosmetics, particularly lipstick, has historically enhanced facial appeal. This study evaluates laypeople's perceptions of the attractiveness and employability of two female models from different age groups, each with varying malocclusions, both with and without lipstick.

**Materials and Methods:**

This cross‐sectional study involved 77 lay participants who assessed digitally edited photographs of young and older female models with varying malocclusions (IOTN 1, 3 and 5), each shown with and without red lipstick. Eye‐tracking technology and questionnaires were used to evaluate perceptions of attractiveness and employability. Data were analysed using one‐way ANOVA and post hoc tests, and Pearson's chi‐squared test.

**Results:**

IOTN 1 with lipstick was rated 49.71 ± 25.74 and without lipstick 47.88 ± 25.44. These were significantly higher than IOTN 3 with lipstick (40.62 ± 24.16; *p* = 0.015), IOTN 3 without lipstick (39.23 ± 23.85; *p* = 0.002), IOTN 5 with lipstick (34.25 ± 24.77; *p* < 0.001) and IOTN 5 without lipstick (31.39 ± 23.30; *p* < 0.001). Employability ratings also varied significantly when comparing IOTN 1 and IOTN 5 images (*p* < 0.001). Eye‐tracking heat maps revealed that the mouth was the primary area of visual focus across all conditions, regardless of lipstick use.

**Conclusion:**

Malocclusion significantly impacts visual attention and attractiveness perception, with the mouth being the primary focus. While lipstick influences attention dispersion, it does not shift the main focus from the mouth. Heat map analysis confirmed that the mouth area remains the primary focus across all images, regardless of lipstick use.

## 1 Introduction

Lips play an important role in the perception of human beauty, and their appearance contributes significantly to an individual's facial attractiveness. A smile is a key component of facial expression and is fundamental to enhancing self‐esteem and overall appeal [1]. As a result, cosmetic treatments aimed at improving the smile are highly sought after, particularly by younger individuals. This demand is strongly influenced by the widespread presence of social media, which amplifies beauty standards and visual self‐presentation [2].

The pursuit of facial beauty is a longstanding phenomenon. From ancient Greece to modern times, women have used facial makeup as a means to enhance their appearance, with the belief that cosmetics increase attractiveness [3]. This historical trend continues today, with makeup serving not only to influence how others perceive us but also to improve self‐perception and self‐confidence [4]. Among various cosmetic strategies, products that emphasise the lips and eyes—such as lipstick—are particularly effective in accentuating facial features that are culturally and evolutionarily associated with attractiveness [3].

Facial attractiveness is shaped by both structural and soft tissue features. In females, large eyes, smooth skin, fuller lips and a harmonious smile are commonly associated with beauty, while in males, defined jawlines, prominent cheekbones, and overall facial symmetry are often considered attractive [4, 5]. Symmetry, averageness and specific facial proportions contribute to what is perceived as a beautiful face [5]. The smile, in particular, plays a critical role not only in aesthetic evaluation but also in social interactions, as it conveys emotions such as confidence and approachability [6]. A pleasant smile can positively affect first impressions and influence social judgements in both personal and professional contexts [6].

Designing a smile that integrates harmoniously with surrounding facial structures is essential in aesthetic planning [6]. Lipstick, as a universally accessible cosmetic tool, plays a prominent role in enhancing the smile by drawing attention to the lips [7]. Therefore, features such as lip thickness and lipstick colour should be considered alongside dental elements, such as tooth shape and shade, when planning aesthetic treatments. Studies have shown that the integration of lip and tooth characteristics influences the perceived attractiveness of the smile, with thick or medium lips and reddish hues rated as the most appealing by both laypeople and dental students [8]. However, although cosmetic elements such as lipstick and lip morphology can influence perceived aesthetics, their consideration is still underrepresented in orthodontic diagnosis and treatment planning.

In dental research, eye‐tracking technology has become a valuable tool for investigating visual attention and aesthetic perception. It has been applied to evaluate a range of factors including smile aesthetics, dental alterations, facial attractiveness and the impact of malocclusions [9, 10]. Additionally, the Index of Orthodontic Treatment Need (IOTN) is commonly used due to its simplicity, reproducibility and efficiency in assessing malocclusion severity [11]. Therefore, the aim of this study was to evaluate laypeople's perceptions of attractiveness and employability when viewing images of two female models from different age groups, each digitally modified to display various types of malocclusions, both with and without the use of lipstick.

## 2 Material and Methods

This is a cross‐sectional observational study conducted through eye‐tracking and questionnaires between 5 October and 1 November 2024. The present study received approval from the university ethics committee under number 2235302.

### 2.1 Photography Preparation

**FIGURE 1:**
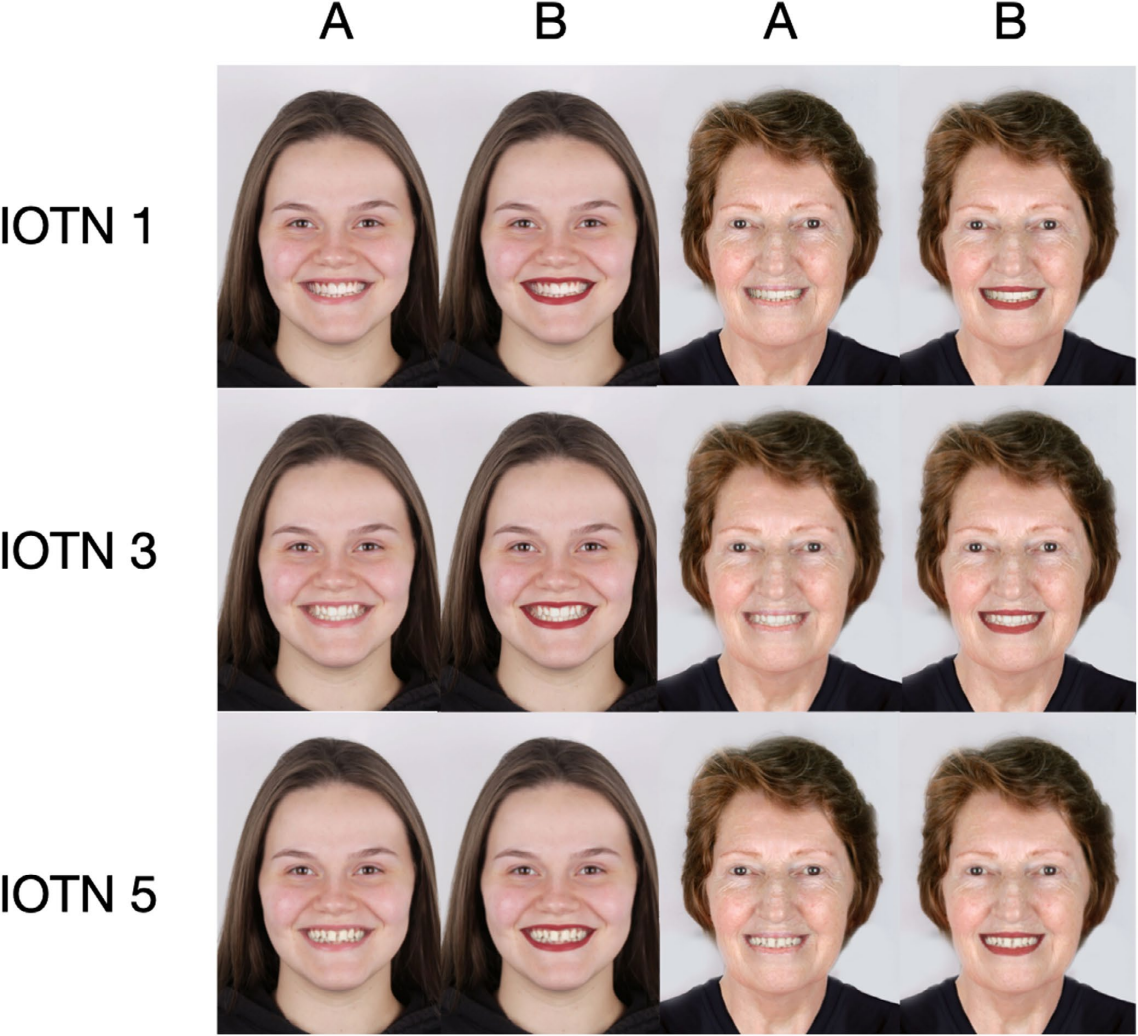
IOTN and models (A) without lipstick, (B) with lipstick.

For this study, two standardised frontal photographs of smiling female subjects, one young adult and one middle‐aged to elderly adult, were selected by a panel of three orthodontists with over 5 years of clinical experience. The original images were chosen based on neutral expression, natural lighting and facial symmetry. Minor skin imperfections, such as blemishes or scars, were removed using Adobe Photoshop CS5 (Adobe Systems, San Jose, CA, USA) to ensure visual consistency across all conditions. Each original image was digitally altered using Photoshop to simulate progressively more severe malocclusions based on the Index of Orthodontic Treatment Need (IOTN). Aesthetic component Specifically, three versions were created for each model: (IOTN): 1: control group (little or no need for treatment), 3: (presence of crowding) and 5: (presence of a diastema). The malocclusions were generated by carefully modifying the anterior dental region while preserving all other facial features, allowing for direct comparison across conditions. Thus, each model's image sequence presented a progressive worsening of malocclusion (Grades 1 → 3 → 5) while maintaining consistency in expression and head position. (Figure 1) In addition, for each malocclusion level, two versions were created: one with red lipstick and one without lipstick. The lipstick was digitally applied using Photoshop tools to ensure realistic shape, colour and shine. The choice of lipstick colour was based on other studies that evaluated different colours such as blue, pink, yellow and orange, and concluded that the colour red was rated as the most attractive [8, 12].

### 2.2 Participants

The participant group for this study consisted of 77 individuals, with 37 males and 40 females, averaging 19.95 years of age. The sample was based on previous studies [13, 14]. Inclusion criteria required participants to be over 18 years old, have no formal education in dentistry, be unaware of the study's objectives, have good vision (with corrective glasses if necessary) and have no prior experience with eye‐tracking studies. To ensure reliable results, exclusion criteria included individuals with neurological disorders, drug or alcohol users, and those taking medications that could impact cognitive abilities.

### 2.3 Data Collection

Eyetracking was conducted using TheEyeTribe hardware in conjunction with Ogama software. Those software have their reliability published [15, 16]. Each image was presented randomly, and the software recorded the number of fixation points and their duration (in milliseconds). To ensure reliable data collection using the OGAMA software, a standardised calibration procedure was performed for each participant prior to the eye‐tracking task. Calibration aimed to reach a ‘perfect’ rating according to the software's internal scale. In cases where the initial calibration did not meet this threshold, the process was repeated, up to three times if necessary, until at least a ‘good’ rating was achieved. Only data from participants who met this minimum calibration standard were included in the analysis.

After selecting the participants and obtaining their signed consent, they were comfortably positioned at a distance of 60–90 cm from the hardware for calibration. Each image was displayed for 5 s on a vertically oriented Dell P2317 monitor, presented in random order. Eye‐tracking data, including heat maps and gaze trajectories, were generated. The heat map indicated the most observed areas within the AOIs, using a colour scale ranging from cool to warm tones (green to red, respectively). Warmer colours indicated a higher number of fixations in that area, while other regions were categorised as ‘other’ and provided additional data. Following the eye‐tracking stage, each participant completed a questionnaire, administered digitally via computer, smartphone, or tablet. For each image, one question assessed attractiveness using the Visual Analogue Scale (VAS): (1) How attractive do you think this person is? (0—not attractive and 100—very attractive) and (1) Would you consider hiring this person?(yes, no, not possible to say just through image).

### 2.4 Statistical Analysis

The results obtained from the eye‐tracking software and questionnaires were tabulated in Microsoft Excel and analysed using the Statistical Package for the Social Sciences version 25 (SPSS; SPSS Inc., Chicago, IL). A one‐way analysis of variance (ANOVA) was applied to identify significant differences between images when the data were normally distributed. Levene's homogeneity test was used to determine whether the distribution was homogeneous or heterogeneous. Post hoc testing was conducted to identify statistical differences; for a homogeneous population, Tukey's honestly significant difference test was used, and for a heterogeneous population, the Games‐Howell test was applied. Pearson's chi‐squared test was employed to compare the different IOTNs with the variables of perception of employability.

### 2.5 Reliability

To assess measurement error, 20% of the image sample was randomly selected and re‐evaluated by the same raters after a three‐month interval. Intra‐rater reliability was calculated using the Intraclass Correlation Coefficient (ICC), yielding values between 0.82 and 0.91, indicating good to excellent agreement. Inter‐rater reliability was also assessed using ICC, with values ranging from 0.78 to 0.89. To evaluate the presence of systematic error, paired measurements were compared using the Student's t‐test. Although these repeated measurements were performed 3 months after the initial evaluations, all procedures were conducted by the same raters under identical conditions and using the same protocols. To minimise the risk of recall bias, the raters were blinded to their initial assessments, and the sample size was sufficiently large to reduce the likelihood of memorisation. Additionally, no training sessions or methodological changes occurred during this period. Therefore, the time interval between assessments did not compromise the reliability of the measurements or the validity of the statistical comparison.

## 3 Results

**FIGURE 2:**
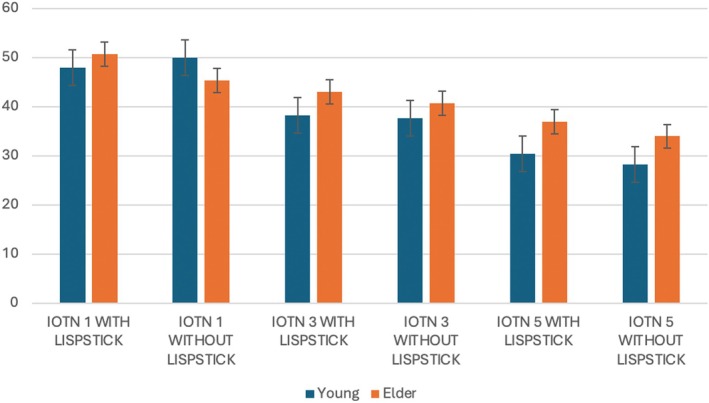
Chart with MEAN Attractiveness Scores.

A total of 77 laypeople participated in the study, with a mean age of 19.5 ± 2.3 years (range: 18–30). The sample included 40 females (mean age: 19.7 ± 2.1 years; range: 18–29) and 37 males (mean age: 19.3 ± 2.5 years; range: 18–30). The mean scores are presented in Figure 2; as for IOTN 1, the highest mean scores were observed in both age groups, with values of 48.2 ± 3.2 (young) and 50.6 ± 2.9 (older) for images with lipstick, and 50.1 ± 3.5 (young) and 45.9 ± 2.7 (older) for images without lipstick. For IOTN 3, scores declined, with younger raters assigning lower mean values (39.2 ± 3.8 with lipstick and 38.7 ± 3.3 without) compared to older raters (43.5 ± 3.1 and 40.6 ± 2.8, respectively). The lowest attractiveness ratings were given to IOTN 5 images, particularly by young raters, who assigned mean scores of 30.4 ± 4.1 (with lipstick) and 28.3 ± 3.9 (without lipstick), while older raters provided slightly higher scores: 36.7 ± 3.4 and 34.2 ± 3.1, respectively.

Notably, attractiveness was higher for models with IOTN 1, regardless of lipstick usage. Significant differences were found when comparing the results of images with and without lipstick: IOTN 1 with lipstick showed significant differences compared to IOTN 3 with lipstick (*p* = 0.015), IOTN 3 without lipstick (*p* = 0.002), IOTN 5 with lipstick (*p* < 0.001) and IOTN 5 without lipstick (*p* < 0.001). Additionally, IOTN 3 with lipstick showed significant differences compared to IOTN 5 without lipstick (*p* < 0.001) (Table 1, Data S1). When comparing data within the same IOTN category, no statistical differences were found between models with and without lipstick (Table 2A). Regarding employability, a statistical difference was observed when comparing IOTN 1 with and without lipstick to IOTN 5 with and without lipstick (*p* < 0.001) (Table 2B). The mean data indicate that most participants responded that it is impossible to determine employability chances based solely on images. Furthermore, there was no statistical difference when comparing employability chances between young and older models (*p* = 0.553) (Data S2), nor in attractiveness (*p* = 0.70) (Table 3), although the older model had a higher mean score.

**TABLE 1:** Attractiveness Scores evaluated with ANOVA.

	Mean	Standard deviation	*p*
IOTN 1 without lipstick	47.88	25.442	*p* < 0.001
IOTN 1 with lipstick	49.71	25.740	
IOTN 3 without lipstick	39.23	23.849	
IOTN 3 with lipstick	40.62	24.159	
IOTN 5 with lipstick	34.25	24.772	
IOTN 5 without lipstick	31.39	23.305	

*Note:* Statistical difference when *p* < 0.05.

**TABLE 2:** Employability chances (A) IOTNs assessed with Pearson Chi‐Square; (B) models age.

			(A) IOTNS						*p*
			IOTN 1 with lipstick	IOTN 1 without lipstick	IOTN 3 with lipstick	IOTN 3 without lipstick	IOTN 5 with lipstick	IOTN 5 without lipstick	
Employability	Yes	Counting	69_a_	70_a_	62_a,b_	58_a,b_	50b	44_b_	*p* < 0.001
		% em IOTNS	43.9%	44.9%	40.3%	37.7%	32.1%	28.0%	
	No	Counting	5_a_	4_a_	9_a,b_	8_a,b_	23_b_	22_b_	
		% em IOTNS	3.2%	2.6%	5.8%	5.2%	14.7%	14.0%	
	Impossible to say just with image	Counting	83_a_	82_a_	83_a_	88_a_	83_a_	91_a_	
		% em IOTNS	52.9%	52.6%	53.9%	57.1%	53.2%	58.0%	

			(B) Models age			
			Young	Elder	Total	*p*
Employability	Yes	Contagem	169_a_	184_a_	353	
		% em Employability	47.9%	52.1%	100.0%	
	No	Contagem	37_a_	34_a_	71	
		% em Employability	52.1%	47.9%	100.0%	0.593
	Impossible to say just with image	Contagem	261_a_	249_a_	510	
		% em Employability	51.2%	48.8%	100.0%	

*Note:* (A) Each subscript letter indicates a subset of Yes or No categories whose column proportions do not differ significantly from each other at the 0.05 level. (B) Each subscript letter indicates a subset of Young or Elder categories whose column proportions do not differ significantly from each other at the 0.05 level.

**TABLE 3:** Pairwise comparison within the same IOTNs.

	Mean	Standard deviation	*p*
IOTN 1 with lipstick	49.71	25.74	0.528
IOTN 1 without lipstick	47.88	25.442	
IOTN 3 with lipstick	40.62	24.159	0.610
IOTN 3 without lipstick	39.23	23.849	
IOTN 5 with lipstick	34.25	24.772	0.296
IOTN 5 without lipstick	31.39	23.305	

*Note:* Statistical difference when *p* < 0.05.

**FIGURE 3:**
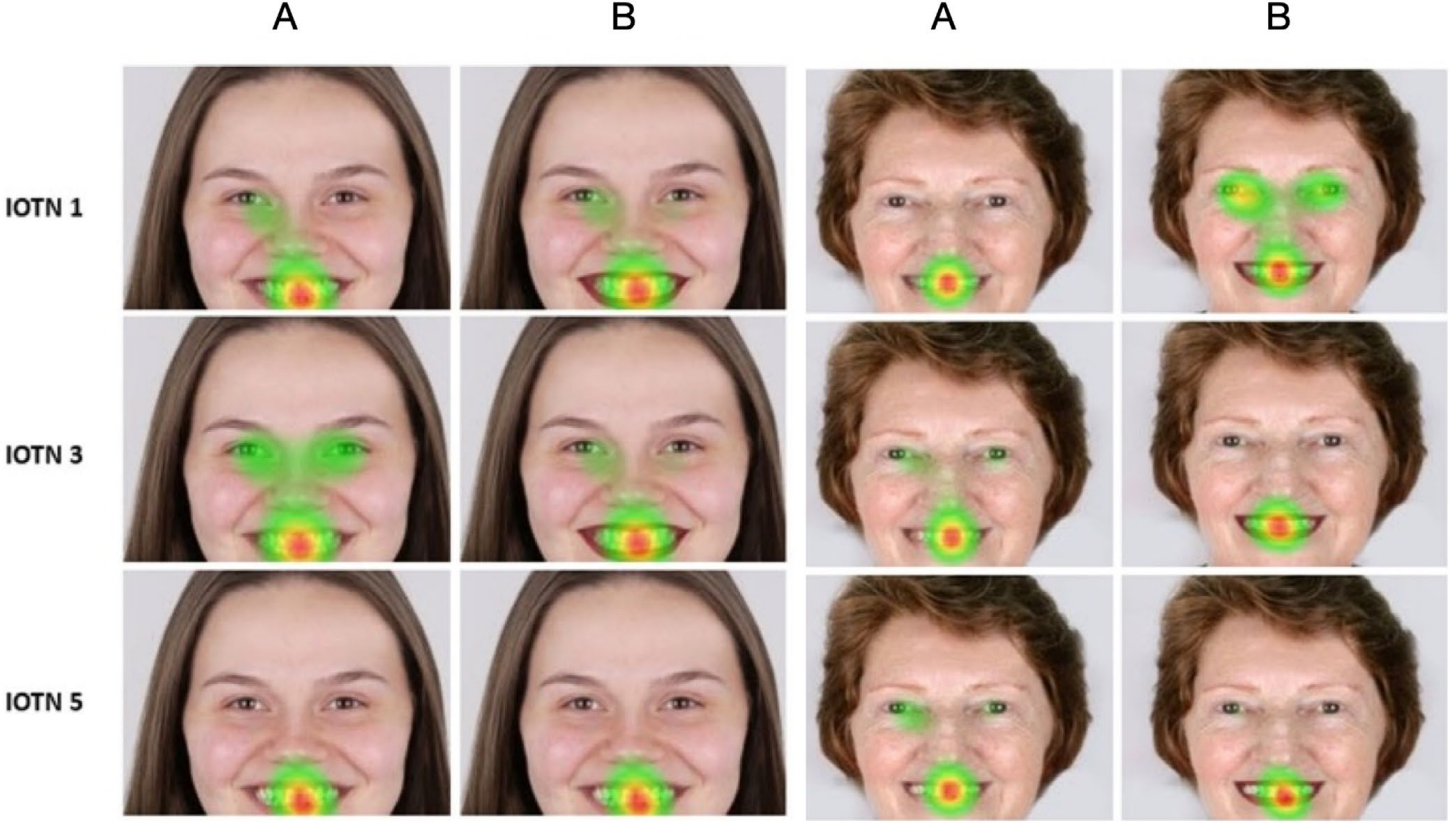
Heatmaps (A) without lipstick, (B) with lipstick.

The heat map analysis from eye‐tracking revealed that, for all images, regardless of the use of lipstick, the main focus was on the mouth area. When examining photographs of the young model with IOTN 1 and 3, both with and without lipstick, participants focused primarily on the mouth region, with some attention also given to the eye region. For photographs with IOTN 5, whether or not lipstick was used, observers' attention was entirely focused on the mouth region, with no dispersion to the eyes, suggesting that the malocclusion was the primary focus of attention regardless of the use of lipstick. For the elderly model with IOTN 1, the mouth was the main focus in photos without lipstick, whereas with lipstick, there was greater dispersion of attention to the nose and eyes. In images with IOTN 3 and 5, attention was primarily on the mouth in photos with lipstick, while in photos without lipstick, there was slight dispersion to the nose and eyes, although the major focus remained on the mouth. When comparing the photographs of the young model with those of the elderly model, it was observed that regardless of the use of lipstick, the primary focus was on the mouth area for all IOTNs. (Figure 3).

## 4 Discussion

In contemporary times, the conception of beauty differs significantly from that of past decades, likely influenced by globalisation and the proliferation of the Internet and social media. These factors continuously modify perceptions of beauty, varying among experts and laypeople, as well as across different parts of the world [17]. The presence of malocclusion affects individuals' self‐esteem, well‐being, and physical and mental health, as well as how they are judged by society [18], individuals with aligned teeth are considered more attractive, intelligent, sociable, and pleasant, honest, and more likely to secure a new job, as well as having credibility, competence and trustworthiness in orthodontists [19], and even increasing their chances of getting a date [20]. Although this study found that models with IOTN 1 were rated more attractive, with significant differences in attractiveness scores when compared to models with IOTN 3 (crowding) and 5 (diastema), regardless of lipstick use. Although no significant differences were found in employability based on images alone or between young and older models.

Eye‐tracking studies have been used in the dental field for a long time due to their ability to identify fixation points and fixation duration, providing better insights into aesthetics. Other studies have been conducted to evaluate dental aesthetics and topics such as diastema, gingival exposure, facial attractiveness, patients with cleft lip and palate, age and malocclusion [9]. In this study, the IOTN was used because it is a quick, simple and easily reproducible method, and because of its efficiency. This method has been used in other similar studies that also utilised eye‐tracking [10]. A study has highlighted the importance of lay opinions on dental aesthetics, considering the divergence between laypeople and professionals in the perception of facial aesthetics. Understanding what laypeople find attractive can help better comprehend patient desires [21]. The heat maps obtained from eye‐tracking revealed that the mouth area was the primary focus across all images, regardless of lipstick use. For the young model, attention was centered on the mouth, especially with higher IOTN scores. The elderly model's photos with lipstick showed more dispersion to the nose and eyes, but the mouth remained the main focus. Overall, malocclusion attracted the most attention, and lipstick influenced attention dispersion but did not shift the primary focus from the mouth.

Regarding malocclusion, IOTN 1, representing little or no need for treatment, was evaluated by participants as the most attractive, with no significant difference between images with and without lipstick. IOTN 3, characterised by crowding, was deemed more aesthetic and attractive compared to IOTN 5, characterised by a diastema. This finding confirms previous study [22, 23], in which no statistical significant difference in aesthetic perception between crowding and diastema. Regardless of the presence of lipstick, the primary focus was on the smile, regardless of the presence of lipstick or not. The results of the heatmaps were consistent with the attractiveness scores, indicating no statistical difference between images with the same IOTN scores with and without lipstick. The difference in attractiveness was perceived between different types of malocclusion.

In this study, digitally manipulated images were used. While digital manipulation allows for controlled and standardised stimuli, it may affect the perceived realism of the images. Previous research has indicated that digitally altered facial images can influence participants' perceptions, potentially introducing bias. For instance, a study found that digitally manipulated images might not accurately represent real‐life conditions [24], affecting the validity of aesthetic assessments. Similarly, alterations in facial features through software could lead to unnatural appearances, impacting observers' judgements [25, 26]. Therefore, while the use of Photoshop in this study facilitated the creation of specific malocclusion scenarios, it is essential to consider the potential impact on the study's ecological validity. Nevertheless, this methodological approach has been widely used in previous studies to simulate and control specific conditions in a standardised manner [11, 13, 14].

This study presents several limitations. Due to the limited sample size relative to the number of potential predictor variables, it was not feasible to perform multivariable regression analyses without risking model overfitting. Also, hiring decisions and assessments of attractiveness should not be based solely on a single image. Additionally, the question ‘Would you consider hiring this person?’ was included as a proxy to explore how visual aesthetics, such as malocclusion or cosmetic use, may influence broader social judgements, including perceptions of professionalism, trustworthiness and competence. While we acknowledge that many participants may not currently be in positions to make hiring decisions, the question was framed hypothetically to reflect social impressions in professional contexts, rather than actual employment choices. However, in many interactions, the first contact is often through photos on social media, where the first impression is crucial. This underscores the importance of considering multiple factors beyond initial visual impressions when making judgements about individuals [23]. Also, the use of lipstick may vary due to cultural aspects, and lipstick is often combined with other makeup, which can soften the contrast between the lips and the teeth. In addition, we acknowledge the lack of standardisation regarding lip thickness; in a study involving dentists, laypeople, and dental students, thicker and medium lips were rated as more attractive than thin lips [8]. The growing demand for aesthetic procedures related to dental and facial aesthetics—such as the popularisation of lip fillers and other facial cosmetic enhancements—along with increased exposure to social media, may be reshaping aesthetic norms. This study highlights the complexity of visual perception and its impact on social judgements, emphasising the need to better understand how various elements interact to shape first impressions. Future studies should consider including dental professionals or orthodontists to compare expert assessments with lay perceptions, as this could provide a more comprehensive understanding of aesthetic evaluation in clinical and social contexts.

## 5 Conclusion

Attractiveness scores were higher for models with IOTN 1, regardless of lipstick use. Employability assessments indicated that models with IOTN 1 had a higher chance of being perceived as employable, independent of lipstick usage. Heat map analysis from eye‐tracking revealed that the mouth area was the primary focus in all images, regardless of lipstick use, with higher IOTN scores attracting more attention. Lipstick influenced attention dispersion to the nose and eyes, particularly in the elderly model, but did not shift the main focus from the mouth.

## Author Contributions

O.T., G.G.G., G.P.Y. were responsible for the literature search, data collection and drafting of the manuscript. M.J.B. and G.G.G., R.A.M. were responsible for the data analysis, data interpretation and discussion. O.T. conceived the idea for the study. G.G.G., O.T., J.S. corrected the manuscript, participated in its design and coordination, and provided the feedback on the revisions to the manuscript.

## Ethics Statement

The study protocol was approved by the Ethics Committee of the university (registration number: 2235302).

## Consent

The authors have nothing to report.

## Conflicts of Interest

The authors declare no conflicts of interest.

## Supporting information


Data S1.

Data S2.

## Data Availability

The datasets used and/or analysed during the current study are available from the corresponding author on reasonable request.
